# Evaluation of the chemical, physical, and biological properties of a newly developed bioceramic cement derived from cockle shells: an in vitro study

**DOI:** 10.1186/s12903-023-03073-0

**Published:** 2023-06-03

**Authors:** Monthip Wannakajeepiboon, Chankhrit Sathorn, Chatvadee Kornsuthisopon, Busayarat Santiwong, Thanakorn Wasanapiarnpong, Pairoj Linsuwanont

**Affiliations:** 1 Department of Operative Dentistry, Faculty of Dentistry, Chulalongkorn University, Bangkok, Thailand; 2 School of Dentistry and Medical Sciences, Charles Sturt University, Parramatta, NSW Australia; 3 Dental Stem Cell Biology Research Unit, Faculty of Dentistry, Chulalongkorn University, Bangkok, Thailand; 4 Department of Pediatric Dentistry, Faculty of Dentistry, Chulalongkorn University, Bangkok, Thailand; 5 Department of Materials Science, Faculty of Science, Chulalongkorn University, Bangkok, Thailand

**Keywords:** Cockle shell, BioCement, Tricalcium silicate, Bioceramic, Dental pulp, Chemical properties, Physical properties, Biological properties

## Abstract

**Background:**

Tricalcium silicate is the main component of commercial bioceramic cements that are widely used in endodontic treatment. Calcium carbonate, which is manufactured from limestone, is one of the substrates of tricalcium silicate. To avoid the environmental impact of mining, calcium carbonate can be obtained from biological sources, such as shelled mollusks, one of which is cockle shell. The aim of this study was to evaluate and compare the chemical, physical, and biological properties of a newly developed bioceramic cement derived from cockle shell (BioCement) with those of a commercial tricalcium silicate cement (Biodentine).

**Methods:**

BioCement was prepared from cockle shells and rice husk ash and its chemical composition was determined by X-ray diffraction and X-ray fluorescence spectroscopy. The physical properties were evaluated following the International Organization for Standardization (ISO) 9917-1;2007 and 6876;2012. The pH was tested after 3 h to 8 weeks. The biological properties were assessed using extraction medium from BioCement and Biodentine on human dental pulp cells (hDPCs) in vitro. The 2,3-bis(2-methoxy-4-nitro-5-sulfophenyl)-5[(phenylamino)carbonyl]-2 H-tetrazolium hydroxide assay was used to evaluate cell cytotoxicity following ISO 10993-5;2009. Cell migration was examined using a wound healing assay. Alizarin red staining was performed to detect osteogenic differentiation. The data were tested for a normal distribution. Once confirmed, the physical properties and pH data were analyzed using the independent t-test, and the biological property data were analyzed using one way ANOVA and Tukey’s multiple comparisons test at a 5% significance level.

**Results:**

The main components of BioCement and Biodentine were calcium and silicon. BioCement’s and Biodentine’s setting time and compressive strength were not different. The radiopacity of BioCement and Biodentine was 5.00 and 3.92 mmAl, respectively (p < 0.05). BioCement’s solubility was significantly higher than Biodentine. Both materials exhibited alkalinity (pH ranged from 9 to 12) and demonstrated > 90% cell viability with cell proliferation. The highest mineralization was found in the BioCement group at 7 days (p < 0.05).

**Conclusions:**

BioCement exhibited acceptable chemical and physical properties and was biocompatible to human dental pulp cells. BioCement promotes pulp cell migration and osteogenic differentiation.

## Background

Regenerative endodontic procedures are biologically-based procedures designed to physiologically induce dentin and root formation, as well as the cells of the pulp-dentin complex [1]. Bioceramic materials are popular in medicine and dentistry. In endodontics, bioceramic cements are used in perforation repair, apexification, vital pulp therapy, and endodontic surgery [2] due to their good biocompatibility, high bioactivity, and antibacterial properties [3].

Tricalcium silicate is the main component of commercial bioceramic cements e.g. ProRoot MTA (Densply Tulsa Dental, OK, USA) and Biodentine (Septodont, Saint-Maur-des-Fossés, France), that are used widely in endodontic treatment. MTA is biocompatible, antibacterial, and induces mineralization [4]. However, it also has a long setting time [5], contains heavy metals [6], discolors teeth [7], and is relatively expensive. Biodentine was developed to address these disadvantages. It has a shorter setting time (15 min) than MTA (170 min) [8] and does not discolor teeth [7]. However, Biodentine is still rather costly.

Traditionally, calcium carbonate is manufactured from limestone, which must be mined. To avoid the environmental impact of mining, calcium carbonate can also be obtained from biological sources, such as mollusk shells, including cockle shells. These shells are discarded in large amounts in the food industry. Cockle shells have a high calcium carbonate content [9]. These shells are abundantly available at a minimal cost, and in vitro studies have demonstrated that the calcium carbonate derived from cockle shells is biocompatible [10]. Nanoparticles derived from cockle shells have been used in medicine as a drug carrier targeting cancer cells [11]. Cockle shells have the potential to be a biological substrate that would lower the financial and environmental cost for manufacturing tricalcium silicate. However, there is no report concerning using cockle shells as a source for calcium carbonate in the preparation of a novel bioceramic cement.

The aim of this study was to evaluate and compare the chemical, physical, and biological properties of a newly developed bioceramic cement derived from cockle shells (BioCement) and Biodentine.

## Methods

### Tricalcium silicate cement preparation and characterization

BioCement is a tricalcium silicate cement prepared from cockle shells and rice husk ash as biological sources for calcium carbonate and silica, respectively. Cockle shells were collected from a local seafood restaurant in Bangkok, Thailand. Rice husk ash was received from A.T. Biopower Power Plant, Pichit Province, Thailand. Cockle shells and rice husk ash were boiled in distilled water for 1 h and then boiled in 5 w% acetic acid solution for 1 h. They were washed twice with distilled water and dried at 105 ^o^C for 24 h. Prepared cockle shells and rice husk ash were mixed with the CaO:SiO_2_ mole ratio of 3:1 to get the tricalcium silicate composition. The mixture was dry ball milled into powder using zirconia planetary mill (Pulverisette 6, Fritsch, Germany) with a speed of 400 rpm for 30 min. The mixed raw powder samples were uniaxial hydraulically pressed (AS ONE NT-100 H Desktop Newton Press 100kN, Japn) at 10 MPa to form 35-mm diameter and 10-mm thick pellets. The pellets were place in an alumina crucible and fired at 1450 °C for 2 h in a high temperature electrical furnace (MoSi_2_ 1700 Heraeus, Germany). The pellets were rapidly cooled using a blower fan. The fired pellets were then crushed in an alumina mortar. Calcined zircon (a radiopacifier) was prepared by firing zirconium silicate powder (ZrO_2_ + HfO_2_ 48%, D_50_ 1.4 micron, Zircosil-1 Plus, Zircosil, Malaysia) at 1000 ^o^C for 1 h in an electrical furnace. The calcined zircon powder was added to the tricalcium silicate cement powder at 20 wt%. The mixture of tricalcium silicate cement and calcined zircon was wet ball milled, with acetone as a medium, using zirconia planetary mill with a speed of 400 rpm for 30 min. After milling, the slurry was dried in an oven at 80 ^o^C overnight. The final powder size was 4.92 micron. The chemical composition of BioCement and Biodentine were characterized. The main chemical constituents were evaluated by an X-ray diffractometer (XRD). The diffractometer (D8 Discover, Massachusettes, USA) used Cu Kα radiation at 40 mA and 45 kV. The detector was set to rotate between 15 and 45°, a sampling width of 0.05° and scan speed of 2° per min. Phase identification was accomplished using a search-match software utilization ICDD database (International Centre for Diffraction Data, PA, USA). The quantitative chemical analysis was performed using X-ray fluorescence spectrometry (XRF). A diffractometer (S8 Tiger, Massachusettes, USA) with Co Kα radiation (1.78 A) was used. The x-ray patterns were acquired in the 2θ(5–60°) with a step of 0.019° and 3 s per step. Phase identification was accomplished using a search-match software utilization ICDD database (International Centre for Diffraction Data, PA, USA).

### Physical properties testing

BioCement was mixed with a 20 wt% calcium chloride solution in a capsule mixer for 30 s. The weight of the solid and solution was 1 and 0.3 gram, respectively. Biodentine (Septodont, Saint-Maur-des-Fossés, France) was prepared by mixing powder and liquid containing calcium chloride accordingly to the manufacturer’s instructions. The physical properties were determined following the International Organization for Standardization (ISO) 9917-1;2007 (Dentistry for water-based cements) and 6876;2012 (Dentistry for root canal sealing materials).

#### Setting time

The setting time was evaluated based on ISO 9917-1;2007 and 6876;2012. Three test specimens for each group were mixed and placed into a rectangular metal block with internal dimensions of 5 × 8 × 10 mm. A Gilmore needle with a 2±0.1 mm diameter tip and a 100±0.5 g and 400±0.5 g force was used to determine the initial setting time and final setting time, respectively. The Gilmore needle was lowered vertically onto the surface of the specimen for 5 s. The process was repeated at 30 s intervals. The time between the end of mixing and the time when the needle failed to make a complete circular indentation on the specimen was recorded as the setting time. The materials were kept in a Temperature and Humidity Controlled Chamber (THCC575, King Mongkut’s Institute of Technology Ladkrabang, Bangkok, Thailand) at 37±1 °C and 95% humidity throughout the analysis.

#### Compressive strength

The test was performed based on ISO 9917-1;2007. Cylinder metal molds with internal dimensions of 6±0.1 mm high and 4±0.1 mm diameter were used to fabricate 15 test specimens of each material. The specimens were kept at 37 °C in a humidified atmosphere for 24 h. Each specimen was placed between the upper plate and lower plate of the Universal testing machine (LLOYD, Ametek, Pennsylvania, USA). The compressive load was applied along the long axis of the specimen with crosshead speed test 0.75 mm per second. The maximum load applied to fracture was recorded as the compressive strength.

#### Radiopacity

Metal molds with an internal diameter of 10±0.1 mm and 1±0.1 mm high were used according to ISO 6876;2012. Three specimens per group were produced and kept at 37 °C in a humidified atmosphere for 24 h. Each sample was placed on a digital imaging plate (Carestream Dental, NY, USA) adjacent to an aluminum step wedge. Radiographs were taken by X-Mind DC (Acteon, Norwich, UK) with 65 kV, 10 mA, and a target film distance of 300 mm for 0.3 s. The gray value of the specimens and the aluminum step wedge were determined using CS 7600 Imaging software. The comparable thickness of the aluminum step wedge in mm was recorded.

#### Solubility

The test was performed following ISO 6876;2012. Polysiloxane molds with internal dimensions of 20±0.1 mm diameter and 1.5±0.1 mm high were used to produce 12 specimens in each group. The specimens were kept at 37 °C and in a humidified atmosphere for 24 h and then weighed (x). Two specimens were placed in the first beaker (beaker A) along with 50 ml deionized water. Beaker A was covered and kept at 37 °C and in a humidified atmosphere for 24 h. Another beaker (beaker B) was weighed (y). The water and specimens in beaker A were poured through filter paper grade 42 (GE Healthcare, Buckinghamshire, UK) into beaker B. Beaker A was washed three times with 5 ml deionized water and the water was poured through the filter paper into beaker B. Beaker B was placed in an oven at 110 °C until the collected water was completely evaporated and then weighed (z). The difference between the final and original weight of beaker B was the amount of cement that dissolved from the specimens. The dissolved specimens and initial sample mass were calculated as the solubility (100 * [z-y]/x).

#### pH

Seven BioCement and Biodentine specimens were produced with polysiloxane molds (20±0.1 mm diameter and 1.5±0.1 mm high). Individual specimens were immersed in 5 ml distilled water. The containers were sealed with plastic wrap throughout the experiment. The pH of each container was measured at 3 h, 6 h, 24 h, 48 h, 1 w, 2 w, 3 w, 4 w, 6 w, and 8 w. The pH meter (CLEAN L’EAU Water Analysis Solutions, Kunling Instruments and Equipment Co., Beijing, China) was used following the manufacturer’s instructions.

### Biocompatibility testing

#### Cell culture

The study’s protocol was approved by the Human Ethics Committee (HREC-DCU 2019-089, ref 002/2020). Three human mature permanent teeth were collected from three different healthy donors. The intact teeth with a healthy pulp were extracted for orthodontic or non-functional reasons with written informed consent. The sample size was determined based on previous studies [12, 13]. The teeth were washed with phosphate buffered saline (PBS). The dental pulp was aseptically removed from the pulp chamber and cut into 1 × 1 mm pieces and placed in 35-mm culture dishes. The explants were placed in growth medium consisting of Dulbecco’s Modified Eagle’s Medium (DMEM, Gibco, USA), 1% L-Glutamine (GlutaMAX-1, Gibco, USA), 100 unit/ml penicillin, 100 µg/ml streptomycin and 250 ng/ml amphotericin B (Antibiotic-Antimycotic, Gibco, USA), and 10% Fetal Bovine Serum (FBS, Gibco, USA). The specimens were incubated at 37 °C in a 5% CO_2_ atmosphere. The growth medium was changed every 2 d. At 7 d, the dental pulp cells were inspected using a light microscope to observe their proliferation and attachment. The dental pulp cells were subcultured when they reached 95% confluence. The dental pulp cells were used between the fourth to sixth passages.

#### Tricalcium silicate cement specimen extracts

BioCement and Biodentine were mixed by the same methods for the physical property tests. The mixtures were placed in polysiloxane molds (7 ± 0.1 mm internal diameter and 2 ± 0.1 mm high: 1.21 cm^2^ surface area) and were kept at 37°C and in a humidified atmospherefor 24 h. The specimens were sterilized in an autoclave (121 ^o^C, 15 min) and ultraviolet irradiation for 30 min. Growth medium was added to the specimens and incubated for 24 h at 37°C with 5% CO_2_ following ISO 10993-12. The extraction media was collected and passed through a 0.22 µm filter. Subsequently, the specimen’ extraction media was stored at -20 °C until used for the indirect contact biocompatibility test.

For the Alizarin red staining assay, the bioceramic materials were immersed in osteogenic medium (OM). The OM was prepared by adding 50 µg/ml ascorbic acid (Sigma-Aldrich, MO, USA), 100 nM dexamethasone (Sigma-Aldrich, USA), and 5 mM β-glycerophosphate (Sigma-Aldrich, USA) into the growth medium [14]. The extraction medium in OM was prepared in the same manner as the growth medium.

#### Cytotoxicity assay and cell proliferation

A cytotoxicity assay was performed using a 2,3-bis(2-methoxy-4-nitro-5-sulfophenyl)-5 [(phenylamino)carbonyl] -2 H-tetrazolium hydroxide (XTT) assay (XTT Cell Viability Kit, Cell Signaling technology, USA), according to ISO 10993-5;2009. The XTT assay is based on measuring the cells’ viability via mitochondrial dehydrogenase. The XTT reagent (a slightly yellow compound) reduces mitochondrial dehydrogenase to a water-soluble formazan product (bright orange). The color intensity determined by photometric measurement is used to measure cell cytotoxicity. The human dental pulp cells (hDPCs) (1 × 10^4^ cells per well) were seeded into 96-well plates and maintained in growth medium for 24 h. After 24 h, cells were incubated with the material’s extraction medium without phenol red for 24, 48, and 72 h. Fifty µl XTT was then added to each well for 4 h. The absorbance was measured at 450 nm using a spectrophotometer (Bio-Tek Epoch II, VT, USA). Each condition was analyzed in triplicate. The relative cell viability of the testing material was calculated using the equation:

Relative cell viability of the test material (%) = (Optical density value of the test material/Optical density value of the positive control) x 100.

Cell proliferation was assessed by the absorbance and the 48 and 72 h measurements were normalized to the 24 h measurement.

#### Wound healing assay

The hDPCs (2 × 10^5^ cells per well) were seeded into 6-well plates in growth medium until confluent. A scratch was performed using a sterilized pipette tip. The cells were then exposed to the material’s extraction medium and incubated for 48 h to allow cell migration into the scratch wound area. Images were obtained at 0, 24, and 48 h at the same location using an inverted phase contrast microscope (Olympus, TN, USA). The x- and y-axis values on the initial measurement location were recorded to allow for measuring at the same location at 24 and 48 h. The images were analyzed using ImageJ software (NIH, MD, USA). All samples were performed in triplicate.

#### Alizarin red staining

In vitro mineralization was performed using alizarin red staining. The hDPCs (2 × 10^4^ cells per well) were seeded into 24-well plates and maintained in OM for 24 h. After 24 h, the media were replaced with the extraction OM and cultured for 7 and 14 d. After 7 and 14 d, the cells were fixed with formalin and gently rinsed with deionized water. The cells were incubated with a 2% Alizarin Red S solution (Sigma-Aldrich, CA, USA) for 3 min. The excess stain was washed with deionized water three times. Images were obtained using an inverted phase contrast microscope (Olympus, TN, USA). The stain was solubilized with 10% cetylpyridinium chloride monohydrate (Sigma-Aldrich, MO, USA) solution and the absorbance was measured at 570 nm using a microplate reader (Biotek ELX800, NJ, USA). Each condition was performed in triplicate.

### Statistical analysis

The data were tested for a normal distribution. Once confirmed, the physical properties and pH data were analyzed using the independent t-test, and the biological property data were analyzed using one way ANOVA and Tukey’s multiple comparisons test at a 5% significance level. The statistical analyses and graphs were done using Prism 9 (GraphPad Software, CA, USA).

## Results

### XRD

**Fig. 1:**
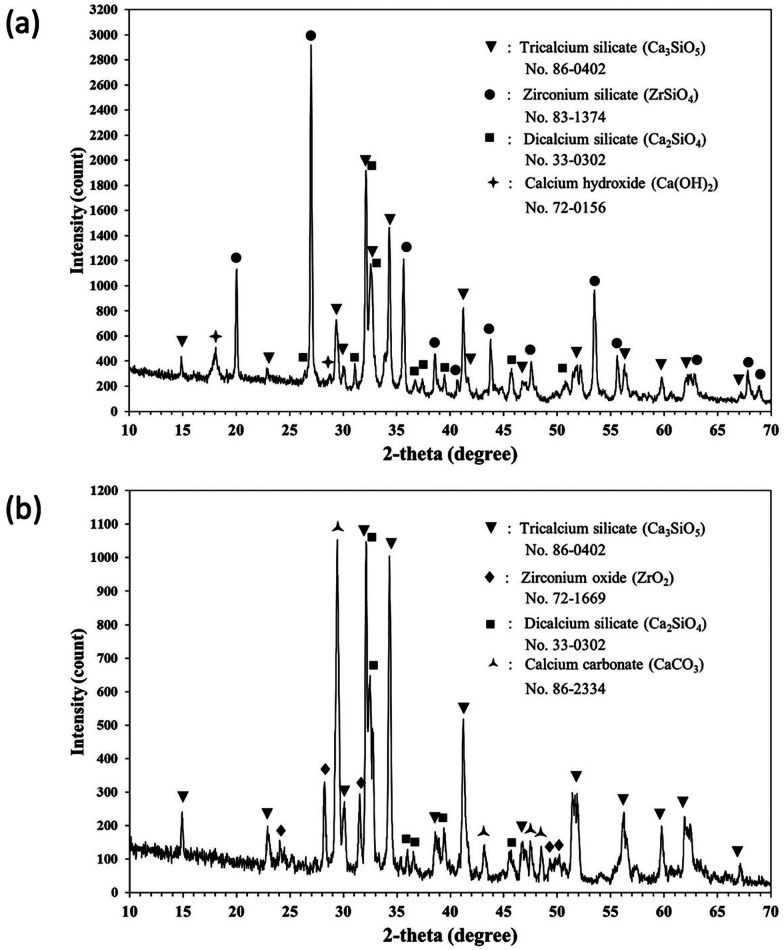
XRD analysis of BioCement and Biodentine (**a**) main components of BioCement, (**b**) main components of Biodentine

The XRD analysis indicated that tricalcium silicate was the main component of BioCement and Biodentine. In addition, Zirconium silicate and zirconium oxide was detected in BioCement and Biodentine, respectively (Fig. 1).

### XRF

The XRF evaluation revealed the chemical composition of BioCement and Biodentine (Table 1). The main elements in BioCement and Biodentine were calcium, silicon, and zircon. However, BioCement had approximately 3-fold more zircon than Biodentine. Both materials had small amounts of magnesium, iron, strontium, and hafnium. Furthermore, sodium, aluminium, manganese, and yttrium were found only in BioCement.

**Table 1:** Chemical composition of Biodentine and BioCement analyzed by XRF. (Proportion by mass)

Composition (%)	CaO	SiO_2_	ZrO_2_	Na_2_O	HfO_2_	Al_2_O_3_	SrO	Fe_2_O_3_	MgO	MnO	Y_2_O_3_
Biodentine	70.60	20.20	5.51	-	0.08	-	0.01	0.09	0.15	-	-
BioCement	58.20	25.20	15.60	0.19	0.16	0.25	0.14	0.11	0.12	0.03	0.03

### Physical properties

We investigated the physical properties of BioCement and Biodentine (Table 2). The initial setting time was 14 min in both groups. However, when loaded with 400 g, the final setting time was 18 and 20 min for Biodentine and BioCement, respectively. The compressive strength of BioCement (55 MPa) was not significantly different from that of Biodentine (53 MPa). Furthermore, BioCement had significantly higher radiopacity values than Biodentine (p < 0.05) of 5.0 and 3.9 mmAl, respectively. In addition, the solubility of BioCement was significantly higher compared with Biodentine (p < 0.05).

**Table 2:** Physical properties of Biodentine and BioCement

Materials	Initial setting time (min)	Final setting time (min)	Compressive strength (MPa)	Radiopaque (mm of Al)	Solubility (%)
Biodentine	14.94$\pm$0.92	18.78$\pm$1.07	53.98$\pm$5.80	3.92$\pm$0.52^a^	3.67$\pm$0.06^a^
BioCement	14.45$\pm$0.25	20.33$\pm$0.44	55.75$\pm$5.71	5.00$\pm$0.14^b^	5.99$\pm$0.08^b^

The different superscripts (a, b) in the same column indicates statistically significant differences between groups (p < 0.05; independent *t*-test)

### pH

**Fig. 2:**
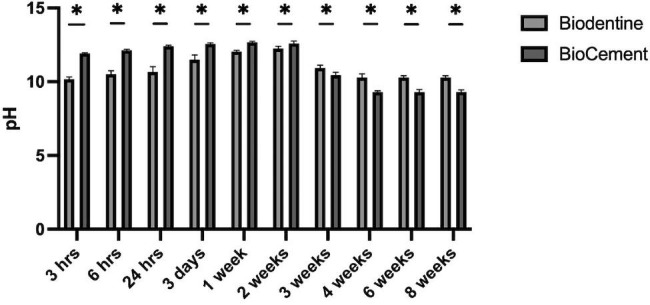
pH of the aqueous medium exposed to Biodentine or BioCement at different time points Asterisk indicates a significant difference between the groups (p < 0.05)

The pH values of the aqueous medium exposed to Biodentine and BioCement at different time points are illustrated in Fig. 2. Both mediums demonstrated alkaline pH values at each time point. The pH value continuously increased, reaching their peak values (approximately pH 12) at 1–2 weeks, decreased, and was stable at 4–8 weeks. At the early stage (3 h to 2 weeks), BioCement had higher significantly pH values compared with Biodentine (p < 0.05). At 4 weeks and later, BioCement and Biodentine presented stable pH values of 9 and 10, respectively.

### Cell viability and cell proliferation

**Fig. 3:**
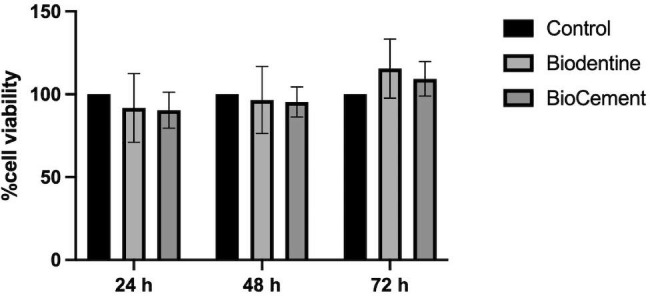
Cell viability in the control, Biodentine, and BioCement groups at 24, 48, and 72 h

**Fig. 4:**
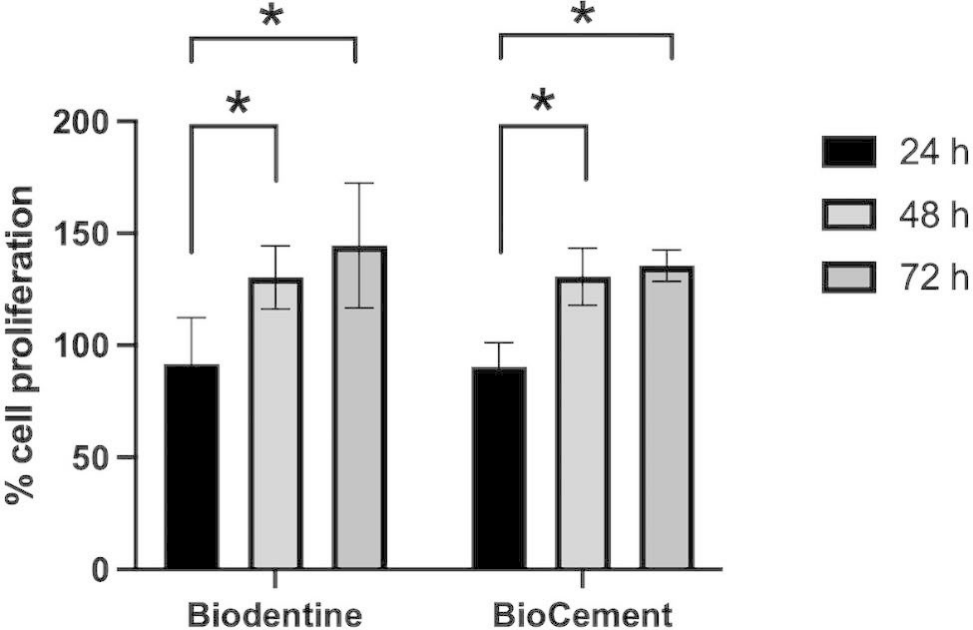
Cell proliferation in the Biodentine and BioCement groups at 24, 48, and 72 h Bars with asterisks indicate a significant difference between the groups (p < 0.05)

The hDPC viability was examined using an XTT assay at 24, 48, and 72 h. Cells in normal growth medium were used as a control. At all evaluated time points, the viability of the hDPCs exposed to the extraction medium from Biodentine and BioCement were not significantly different compared with the control (Fig. 3). The cell viability percentage in the BioCement and Biodentine groups was more than 90%. In addition, the hDPCs exposed to the extraction medium from Biodentine or BioCement proliferated in a time-dependent manner (Fig. 4).

### Cell migration

**Fig. 5:**
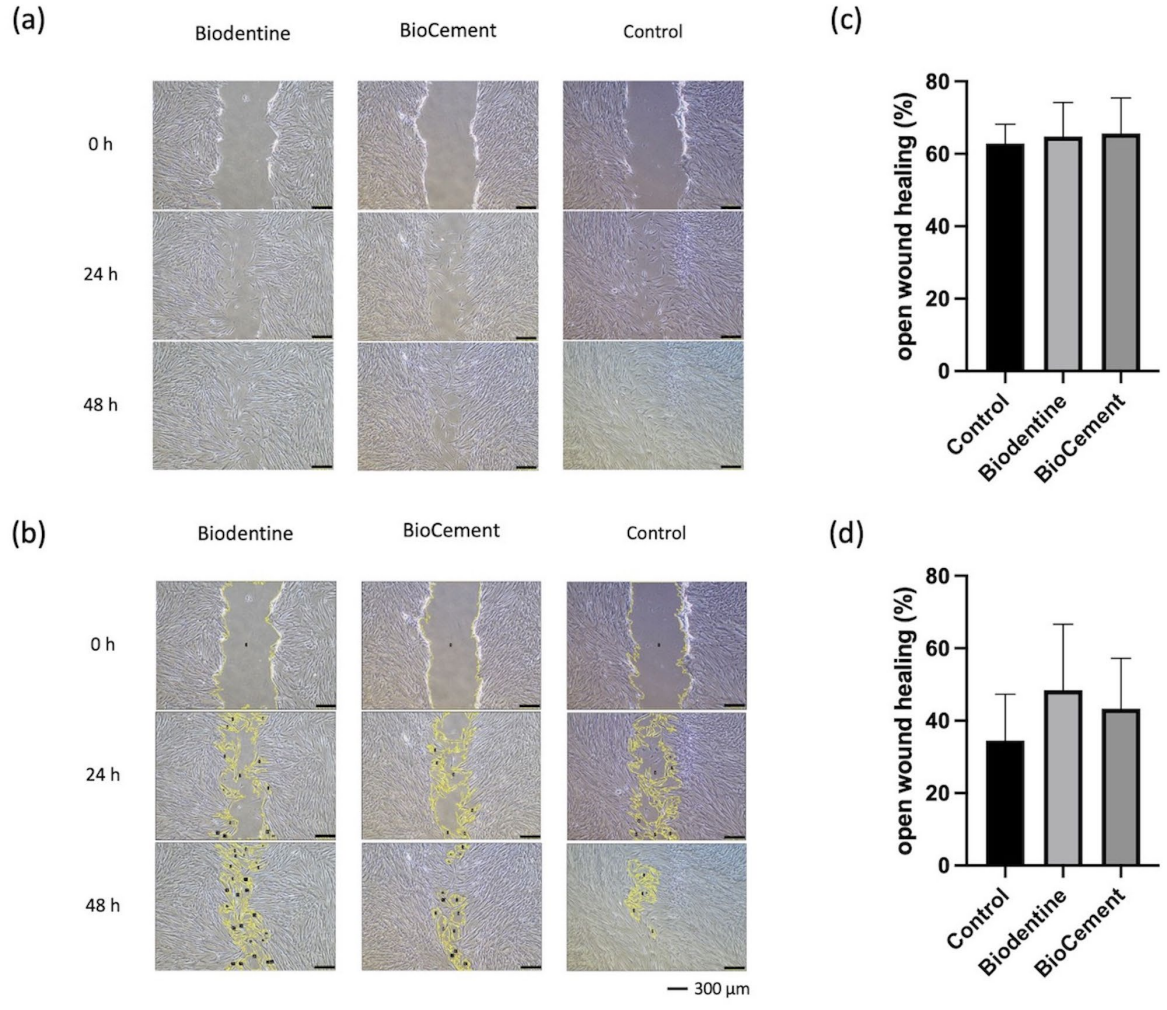
Cell migration evaluation of Biodentine and BioCement in the wound closure simulation (**a**) the scratch areas. (**b**) the scratch areas analyzed by ImageJ. The yellow lines indicate the open wound healing areas. Scale bars: 300 μm. (**c**) percentage of open area at 24 h. (**d**) percentage of open area at 48 h

A wound healing assay was performed to assess cell migration. At baseline (0 h), the images presented a well-defined scratch wound border. Cell migration into the scratched area was observed at 24 and 48 h. At 48 h, the wound area was reduced to 34%, 48%, and 43% in the control, Biodentine, and BioCement, respectively, groups. There was no significant difference in wound area among the control, Biodentine, and BioCement groups at 24 and 48 h (Fig. 5).

### In vitro mineralization

**Fig. 6:**
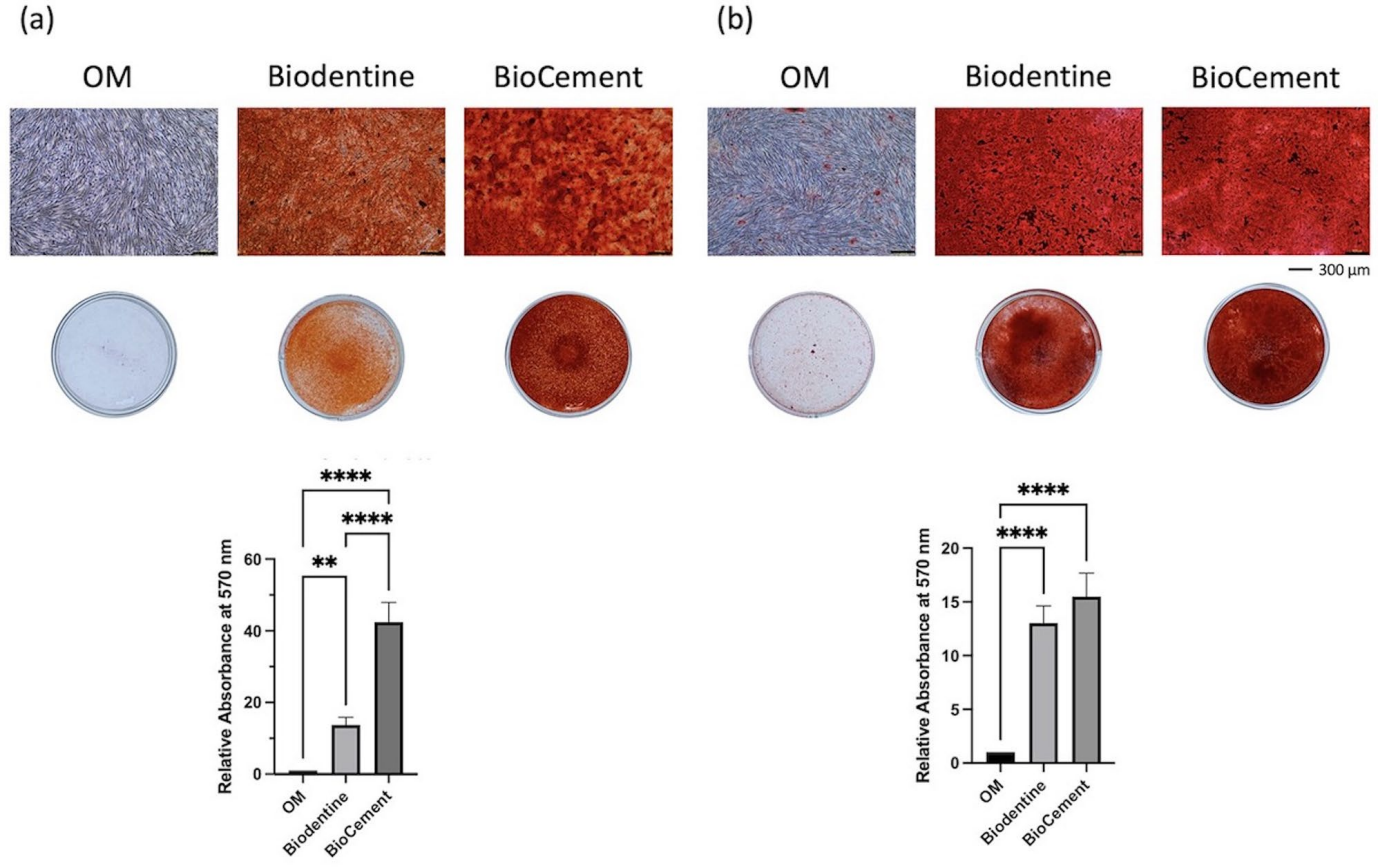
The effects of Biodentine and BioCement on human dental pulp cell osteogenic differentiation (**a**) Alizarin red staining at 7 days. (**b**) Alizarin red staining at 14 days. Scale bars: 300 μm. Bars with asterisks indicate a significant difference between the groups (p < 0.05)

The effect of the materials on hDPC mineralization was investigated using Alizarin red staining (Fig. 6). Cells in OM were used as a control. At 7 days, the cells exposed to the extraction medium from Biodentine or BioCement exhibited significantly increased mineralization compared with the control cells (p < 0.05). The BioCement-treated cells demonstrated the highest mineralization. Similarly, at 14 days, cells exposed to the extraction medium from both materials presented higher mineralization than the cells in OM (p < 0.05). However, there was no significant difference in mineralization between the BioCement and Biodentine groups.

## Discussion

Biodentine is a calcium silicate-based bioceramic developed to address the limitations of MTA. Studies have found that Biodentine is suitable for regenerative endodontics because of its biocompatibility, osteogenic differentiation and, importantly, high clinical success rates [14–18]. Therefore, Biodentine was used in this study as the standard to which BioCement was compared.

There are several ways to prepare tricalcium silicate [19–21], i.e., sol-gel reaction and solid-state reaction, which was used in the present study. Tricalcium silicate was produced from cockle shell-derived calcium carbonate (CaCO_3_) and rice husk ash-derived silica dioxide (SiO_2_), as shown in the equation: CaCO_3_ + SiO_2_ -> Ca_3_SiO_5_ + CO_2_. Calcium chloride was added to accelerate the setting time and zirconium silicate to increase the radiopacity of the material. Zirconium silicate is inert, inexpensive, and readily available. Furthermore, it has low thermal linear expansion [22], and high resistance to thermal shock [23, 24]. Zirconium silicate decreases the likelihood of crack propagation in a material.

The XRD and XRF analysis demonstrated that the main chemical components of BioCement and Biodentine were calcium oxide and silicon dioxide, which are the precursors for tricalcium silicate. Therefore, cockle shells and rice husk ash can be an eco-friendly, low cost source of tricalcium silicate.

The materials for clinical use should have the desirable chemical, physical, and biological properties according to the ISO dental material testing guidelines. The recommended compressive strength for a dental cement is 50 MPa (ISO 9917-1;2007), which is comparable to the observed masticatory force [25]. BioCement and Biodentine both met this standard. In addition, a dental cement should have a radiopacity greater than 3 mm of aluminum (ISO 6876; 2012). The radiopacity of BioCement and Biodentine were both above this benchmark. Furthermore, when cultured with the extraction media of each material, more than 90% cell viability was observed at all observation time points, which surpassed the suggested value of 70% cell viability (ISO 10993-5;2009).

The physical and biological properties of Biodentine found in this study were in agreement with previous studies using ISO guidelines or similar testing methods i.e. setting time [26, 27], compressive strength [26], radiopacity [28–30], pH [31], biocompatibility [14], cell migration [15, 32, 33], and in vitro mineralization [13, 14, 34]. Hence, our results using the various ISO methods can be considered accurate.

Single-visit treatment for regenerative endodontics is possible only if the material has a short setting time. BioCement has an initial setting time of 14 min; therefore, it can be used for this purpose. In vital pulp therapy, a bioceramic cement is placed directly on the pulp wound, and the tooth is restored with the permanent restoration. Thus, a specific level of compressive strength is required to withstand the masticatory forces. The radiopacity of a dental cement is used to differentiate the dental material from the tooth structure. This allows the integrity of the interface between the two to be assessed. BioCement met the ISO standard for compressive strength and radiopacity. Thus, it can be considered for clinical use. The alkalinity of BioCement has an antibacterial effect, and stimulates low-grade inflammation, which promotes tissue healing and hard tissue remineralization [35].

BioCement has a higher solubility than Biodentine (5.9% vs. 3.6%). Previous reports have found a wide range of Biodentine solubility (2.6–13.34%) [36–38], which is higher than 3% as suggested by ISO 6876;2012. The possible reason for this may be variations in testing methods. The higher solubility of BioCement than Biodentine may be partly from the difference of the chemical compositions in the powder and liquid. Bioceramics expand upon setting [39] and this may compensate for the material’s dissolution. Their porosities and voids have been shown to reduce over time [40]. It is likely that with time, the solubility of the tested materials will reduce. In addition, ISO 6876; 2012 is the standard for sealer testing. However, there is no specific ISO for dental cement solubility. The ISO standard of 3% solubility, therefore, may not be directly applicable to dental cements.

There are several ways to test cell cytotoxicity, such as the Neutral red uptake, WST, MTT, and XTT assays [26, 41, 42]. A systematic review revealed that the XTT assay had good concordance with cell cytotoxicity, while the MTT assay had moderate concordance with cell cytotoxicity [42]. Thus, the XTT assay was chosen to assess cell cytotoxicity. To ensure that the biocompatibility assessment is of high quality, it should be evaluated by multiple methods. Although a wound healing assay is typically performed to observe cell migration [15], it was used in this study to measure biocompatibility. In addition to materials for vital pulp therapy being biocompatible, they should also induce hard tissue formation, which indicates complete healing of the pulp. Calcium silicate setting reaction is hydration [43], which lead to calcium hydroxide formation. The released hydroxyl ions upon hydration will increase in pH, while the released calcium ions contribute to protective dentin-bridge formation as they stimulate DPCs differentiation and increase the formation of mineralized nodules. Silicon ions may play in role in dentin-bridge formation by stimulate osteoblast for bone formation [44].

In the present study, Alizarin red staining was used to assess the mineralization effect of our tested materials. Biodentine has been found to induce dentine bridge formation as demonstrated histologically at 6 weeks [45, 46] and radiographically at 9–12 weeks [47]. The in vitro mineralization effect of BioCement was observed in this study at 7 d. However, this is only preliminary evidence of BioCement’s mineralization effect. A longer observation period and in vivo studies would provide stronger evidence of mineralization.

Although BioCements properties met the ISO standards, it should be further evaluated in animal and human studies prior to its clinical use in endodontics especially in the area of vital pulp therapy and revitalization.​.

## Conclusion

BioCement is a tricalcium silicate-based material manufactured from items discarded during food preparation; cockle shells and rice husk ash, which are readily available, inexpensive, and environmentally friendly. According to multiple ISO testing guidelines, BioCement has acceptable chemical, physical, and biological properties similar to those of Biodentine.

## Data Availability

The datasets used during the current study available from the corresponding author on reasonable request.
